# Magendie and Luschka: Holes in the 4^th^ ventricle

**DOI:** 10.1590/S1980-5764-2016DN1003015

**Published:** 2016

**Authors:** Eliasz Engelhardt

**Affiliations:** 1 Cognitive and Behavioral Neurology Unit - INDC-CDA/IPUB - Federal University of Rio de Janeiro (UFRJ), Rio de Janeiro-RJ, Brazil.

**Keywords:** cerebrospinal fluid, median opening, lateral openings, 4^th^ ventricle, Magendie, Luschka

## Abstract

Cerebrospinal fluid (CSF) is a complex liquid formed mainly by the choroid plexuses. After filling the ventricular system where it circulates, CSF flows out to the subarachnoid spaces through openings in the 4^th^ ventricle. Following numerous studies on CSF pathways, these openings were first discovered in the 19^th^ century by two notable researchers, François Magendie and Hubert von Luschka, who described the median and lateral openings subsequently named after them. Even after the studies of Axel Key and Gustav Magnus Retzius confirming these openings, their existence was questioned by many anatomists, yet acknowledged by others. Finally gaining the acceptance of all, recognition of the holes endures to the present day. Interest in these openings may be attributed to the several congenital or acquired pathological conditions that may affect them, usually associated with hydrocephalus. We report some historical aspects of these apertures and their discoverers.

## RESUMO

O líquido cefalorraquidiano é um fluido complexo formado principalmente pelos plexos coroides que, após preencher o sistema ventricular, onde circula, flui para os espaços subaracnóides através de aberturas do 4^o^ ventrículo. Antecedido por numerosos estudos sobre as vias do líquido cefalorraquidiano, estas aberturas foram descobertas pela primeira vez no século XIX graças a dois pesquisadores notáveis, François Magendie e Hubert von Luschka, que descreveram as aberturas mediana e laterais do 4^o^ ventrículo, as quais receberam, então, seus nomes. Apesar dos estudos de Axel Key e Gustav Magnus Retzius que confirmaram a existência de tais aberturas, estas foram postas em dúvida por muitos anatomistas, mas aceitas por outros, para finalmente serem reconhecidas por todos, o que perdura até os presentes dias. O interesse por essas aberturas pode ser atribuído a diversas condições patológicas congênitas ou adquiridas que podem atingi-las, geralmente associadas à hidrocefalia. Aqui são relatados alguns aspectos históricos sobre essas aberturas e seus descobridores.

## INTRODUCTION

Cerebrospinal fluid (CSF), a complex liquid produced mainly by the choroid plexuses, circulates in the ventricular system, runs out through openings of the 4^th^ ventricle, flows into the subarachnoid spaces, to be finally absorbed mostly at the arachnoid granulations in the superior sagittal venous sinus. The discovery of these openings in the 4^th^ ventricle, first described in the 19^th^ century by François Magendie and Hubert von Luschka, resulted from continued and tenacious research on the subject.^1^ ^,^ ^2^ These openings aroused interest for their importance under normal conditions, and in several congenital or acquired pathological disorders, usually associated with hydrocephalus, which may affect them. These conditions include occlusion, membrane obstruction, congenital imperforation, idiopathic stenosis, arachnoid adhesions and cystic dilation, and hamper the normal flow of cerebrospinal fluid.^1^

In this article, some historical aspects of these apertures and the individuals involved in their discovery will be described. 

## MAGENDIE

**Figure 1:**
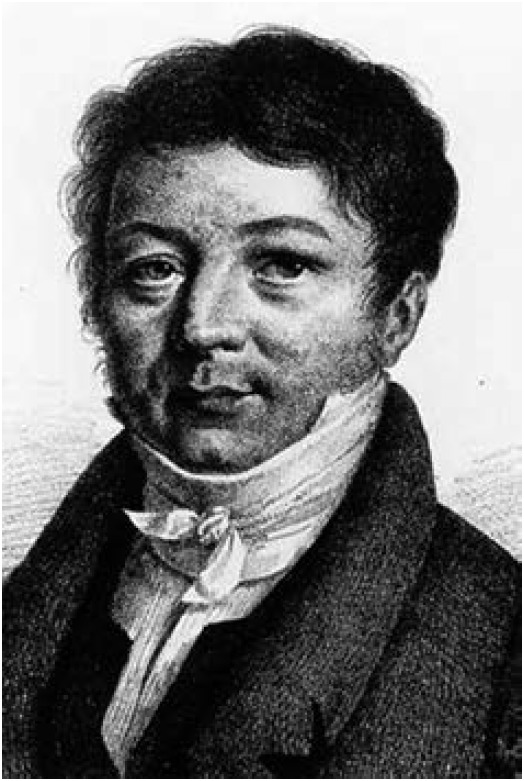
François Magendie - portrait by unknown artist (1822).

François Magendie (1783-1855) (Figure 1) was a French physician, anatomist and physiologist. Among his numerous studies, those on the CSF were impressive, the first of which was published in 1825. In his book "Physiological dissertation on the cerebrum" (*Mémoire physiologique sur le cerveau*) (1828) he recognized the existence of a liquid inside the cranium and spine he denominated, what he called "his" liquid, the "cerebrospinal or cerebrorachidian fluid" (*liquide céphalo-spinal* or *céphalo-rachidien*).^3^ ^,^ ^4^ ^,^ ^7^ Later, in another book, "Physiological and clinical investigations about the cerebrorachidian or cerebrospinal fluid" (*Recherches physiologiques et cliniques sur le liquide céphalo-rachidien ou cérébro-spinal*), with an accompanying atlas (1842), he detailed observations about cerebrospinal fluid, the ventricular system, the subarachnoid spaces, and the opening he discovered communicating these compartments. Also, he performed experimental procedures to study the dynamics of the fluid (Box).^3^ ^,^ ^5^ ^,^ ^6^

From Wikimedia Commons (public domain) https://commons.wikimedia.org/wiki/File:Fran%C3%A7ois_Magendie.jpg

**Box.**
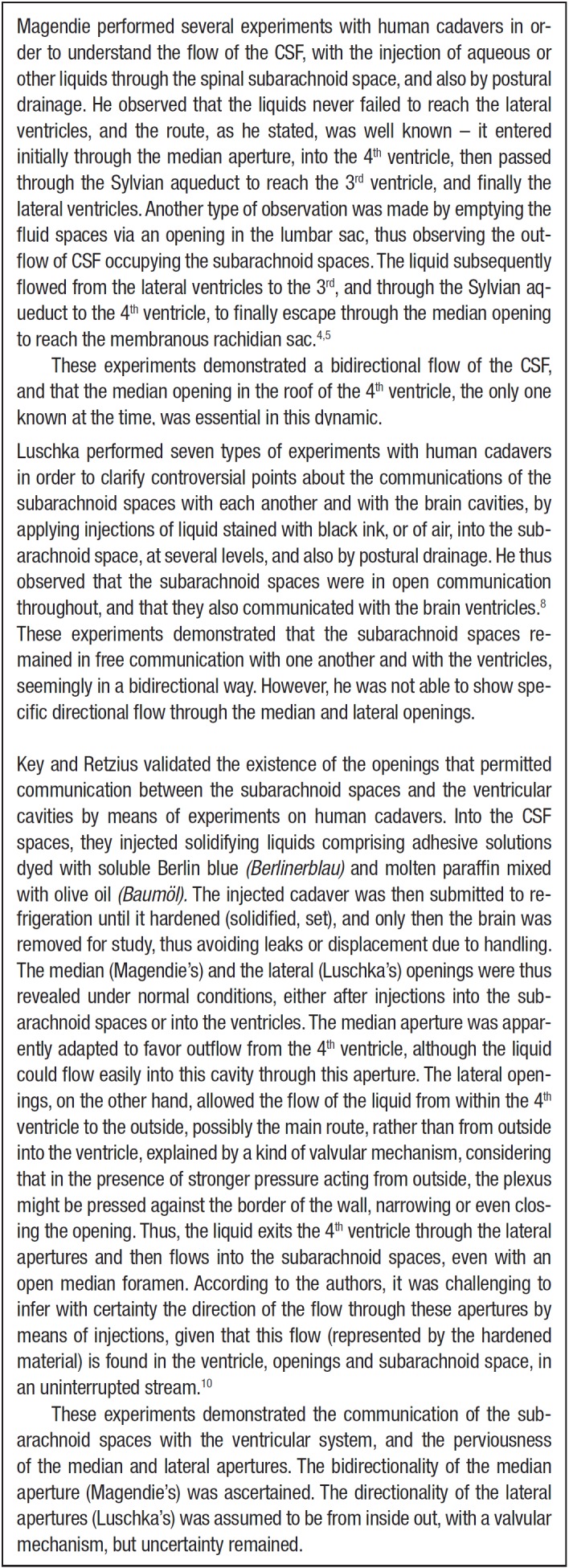
The experimental findings of Magendie, Luschka, and Key & Retzius.

**Figure 2:**
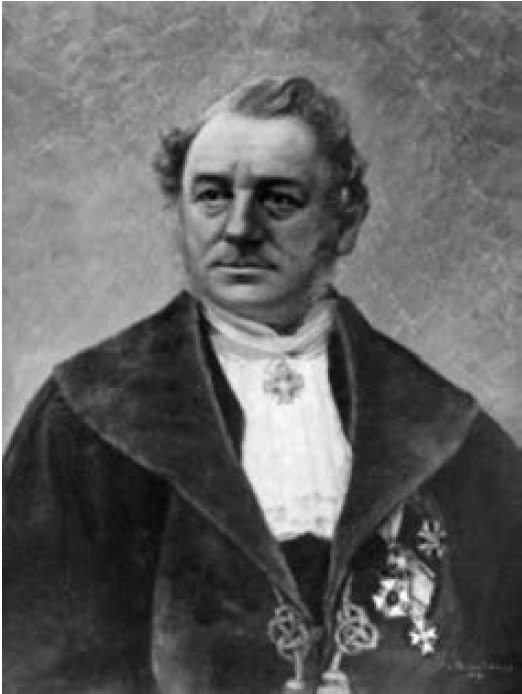
Hubert von Luschka - painting by M. Müller-Schüppel (1896).

Magendie's median opening. Magendie proposed that the fluid present in the subarachnoid spaces and within the ventricles might be the same, writing: "It is understood that, to confirm such conjecture, it would be necessary that an opening existed to establish a communication between the exterior of the organ [brain] and its interior cavities, and such an opening was yet not known", and proceeded: "...indeed, after some more research...I finally found an opening...hidden completely by a lobe of the cerebellum, constituting a true entrance of the cerebral cavities".^4^ He considered the opening remarkable for the direct communication it established between the subarachnoid and ventricular liquids, the location of which he described as follows: "...the constant and normal true opening through which the fluid passes continuously, either to enter the ventricles, or to come out...is seen at the inferior end of the fourth ventricle, near the region named 'beak of the pen (feather)' [*calamus scriptorius*] by the ancient anatomists" *(...la véritable ouverture constante et normale par laquelle passe le liquide céphalo-rachidien constamment, soit pour entrer dans les ventricules, soit pour en sortir...elle se voit à la terminaison inférieure du quatrième ventricule, à l'endroit nommé le bec de la plume par les anatomistes anciens*). He named this aperture "opening of the encephalic cavities" (*orifice des cavités encéphaliques*). Magendie described how to find the opening, its boundaries, and its variable form and size, but illustrated it poorly in a sagittal section of the cerebrospinal axis (Plate 2, Figure 2-b).^5^ ^,^ ^6^

From Wikimedia Commons (public domain) https://commons.wikimedia.org/wiki/File:Hubert_von_Luschka,_Gem%C3%A4lde_von_M._M%C3%BCller-_Sch%C3%BCppel_1896.png

Thus, for the first time, he had uncovered the existence of a median opening of the 4^th^ ventricle that placed this cavity in communication with the subarachnoid spaces, and that was later named after him.^7^ ^,^ ^8^

## LUSCHKA

Hubert von Luschka (1820-1875) (Figure 2) was a German anatomist. In his book "The choroid plexuses of the human brain" (*Die Adergeflechte des menschlichen Gehirns*) (1855), he thoroughly described the cerebral ventricles, subarachnoid spaces, CSF, and the choroid plexuses. He also applied experimental techniques in order to determine dynamic aspects of the fluid (Box).^8^

**Figure 3:**
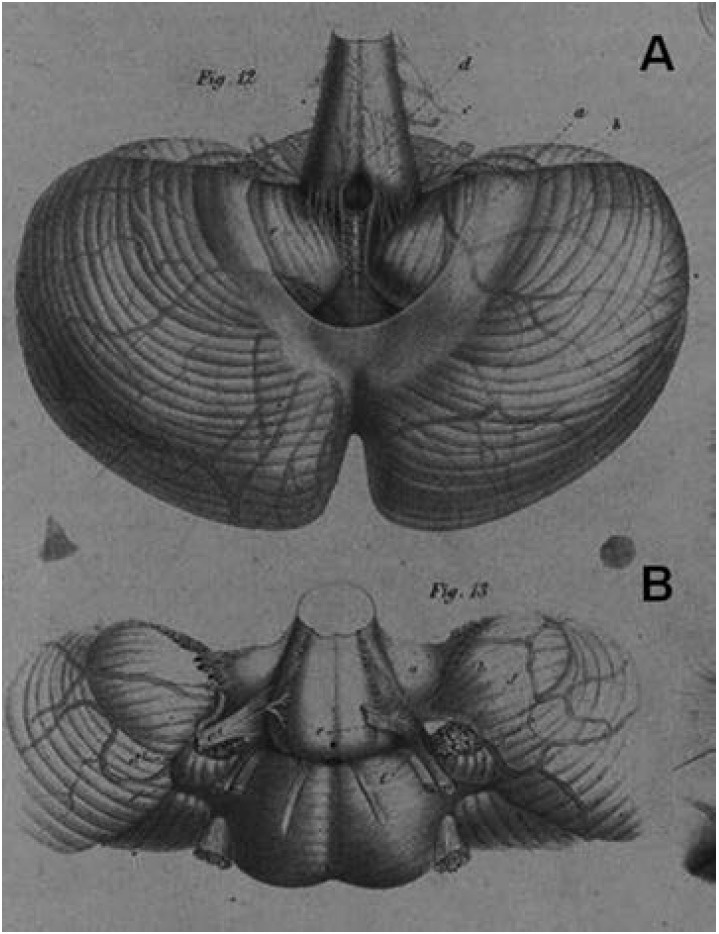
Figures taken from Key and Retzius's Plate III^10^. [A] Figure 12. Cerebellum with medulla oblongata and pons: the medulla oblongata is raised and the arachnoid cutaway in the middle with part of it remaining *(a)*, exposing the cisterna magna and the *Apertura inferior ventriculi quarti* (Magendie's opening) *(d).* [B] Figure 13. Medulla oblongata and pons, and the adjacent part of the cerebellum together with the inferior wall of the 4^th^ ventricle. The vermis was cut across, and both lateral halves of the cerebellum bent forward to demonstrate the *Aperturae laterales ventriculi quarti* (Luschka's openings). To the right side, the inferior wall of the 4^th^ ventricle *(a)* with its anterior border *(b)* can be seen. Between the anterior border and the protruding part of the plexus *(d)*, the *Apertura lateralis* [not labeled] is visible, exposed in full with the glossopharyngeal and vagus nerves *(e)* folded to the side. The left side shows the natural position of the nerves, almost completely covering the opening.

Luschka's lateral openings and more. He gave special significance to the 4^th^ ventricle as a gateway linking the other cerebral cavities with the subarachnoid spaces. He described the boundaries, walls, and angles of the 4^th^ ventricle. According to his report, the inferior part of the lateral wall included the external (lateral) angle [lateral recess] [recessus lateralis, Reichert], on each side, as follows: "The external angle thus bounded extends outwards as a channel (duct), through which protrudes the lateral part of the choroid plexus of the fourth ventricle, while the arachnoid is freely stretched over this location. The external angle places the fourth ventricle in open communication with the subarachnoid space. The opening, where the pia mater merges with the ependymal lining, is walled in such a way by the lateral part of the choroid plexus that only a narrow cleft remains... entirely sufficient to allow a liquid...to emerge..." (*Der so begrenzte äussere Winkel verläuft als eine Rinne nach aussen, durch welche der seitliche Theil des Adergeflechtes der vierten Hirnhöhle heraustritt, während die Arachnoidea über diese Stelle frei hinweggespannt ist. Der äussere Winkel setzt daher den vierten Ventrikel mit dem Subarachnoidealraum in einen offenen Verband. Die Lücke, an welcher die Pia mater in das Ependyma übergeht, ist inzwischen durch den seitlichen Theil des vierten Adergeflechtes so verlegt, dass nur eine enge Spalte übrig bleibt...völlig genügt, um Flüssigkeit...Vorscheine kommen zu lassen*).^8^ He also examined the inferior part of the roof, including the inferior angle of the 4^th^ ventricle, and described: "In the inferior tela choroidea there is an elongated rounded...hole, as first identified by Magendie, which provides the main communication of the brain cavities with the subarachnoid space" (*ln der untern Geläfsplatte befindet sich eine länglichrunde... zuerst von Magendie näher gewürdigte Lücke, welche den hauptsächlichsten Verband der Hirnhöhlen mit dem Subarachnoidealraum vermittelt*). He thus identified the median opening, and acknowledged that Magendie was the first to find and describe an aperture there.^8^ Luschka accurately depicted the median opening (Plate III, Fig. 1-a), but failed to illustrate the lateral ones he so clearly described, representing only (Plate III, Fig. 3) the inferior surface of the cerebellum, where the choroid plexus, comprising its lateral parts, is displayed.^8^

Thus, Luschka described the lateral openings for the first time, a centerpiece of his research and later named after him, and ratified the existence of the median aperture named after Magendie.^8^ ^,^ ^9^

## COMMENTARIES

After a very long period of studies on CSF and its pathways, the openings of the 4^th^ ventricle, which communicate with the two compartments (ventricular and subarachnoid), were discovered thanks to Magendie (median) and Luschka (lateral) and later named after them. It should be pointed out, however, that previous studies on the CSF pathways, some cited below, paved the way for these researchers to achieve their accomplishments.

Axel Key (1832-1901) and Gustav Magnus Retzius (1842-1919),^10^ in a review on history they presented in volume 1 of their book "Studies on the Anatomy of the Nervous System and the Connective Tissue" (*Studien in der Anatomie des Nervensystems und des Bindegewebes*) (1875) recalled that suppositions about passages between the ventricles and the subarachnoid spaces were not envisaged before the studies of von Haller, who described (1747) a space between the pia-mater and the arachnoid membrane, and a fluid on the surface of the brain, considering that such specifics were hitherto unknown. He hypothesized that the ventricular fluid (vapor, liquid) must have a route outwards but without offering any substantiation.^10^ ^-^ ^12^ However, credit must go to Cotugno for the first adequate account of the liquid (1764) present in the ventricles and surrounding the brain and spinal cord, and that could mingle at the level of the fourth ventricle, without providing further details.^10^ ^,^ ^13^ ^,^ ^14^ Bichat described (1799) a distinct channel ending in the third ventricle, located in the tissue around Galen's vein, and establishing a communication between the ventricular serous (arachnoid) membrane and the (external) arachnoid membrane, for the liquid to circulate, claiming anatomic and physiologic evidence.^15^ However, Magendie, and later Key and Retzius, refuted Bichat's findings, considering them artifactitual.^5^ ^,^ ^10^ The issue was settled with Magendie's clear description (1828, 1842) of the subarachnoid spaces (previously reported by Cotugno), and his discovery of a median opening in the roof of the 4^th^ ventricle providing communication of the ventricular with the subarachnoid liquid.^4^ ^,^ ^5^ ^,^ ^8^ Later, Luschka completed understanding on the subject, discovering the lateral apertures of the 4^th^ ventricle, related to the lateral recesses and the protruding parts of the choroid plexus (1855).^8^

The investigations of Key and Retzius^10^ meticulously described the issue (1875). They confirmed Magendie's and Luschka's findings with thorough anatomical descriptions and dynamic experiments, by means of subarachnoid and ventricular injections to demonstrate the perviousness of these openings (Box). They proposed a denomination for the openings - *Apertura inferior* (for the median opening) and *Aperturae lateralis ventriculi quarti* (for the lateral openings), acknowledging the naming after Magendie (according to Luschka) for the median aperture, but maintaining the technical rather than the eponymic denomination for the lateral apertures, and also provided unambiguous illustrations of these openings (Plate III, Figures 12 and 13)^10^ (Figure 3). The clear-cut scientific documentation of the main features of the CSF pathways presented, where the openings in the 4^th^ ventricle play an essential role, have remained valid to the present day.^10^ ^,^ ^16^ Even after confirmatory studies, the existence of these openings was questioned by several anatomists, despite the acknowledgement of many others. Finally, the existence of these openings (holes) was accepted by all researchers, recognition that endures to the present day.^17^
